# Early Life Trajectories of Household Poverty and Area‐Level Deprivation and Childhood Dental Caries: A Longitudinal Data Linkage Cohort Study

**DOI:** 10.1111/cdoe.13051

**Published:** 2025-06-10

**Authors:** Kyle Cousins, Conway David, Paul Bradshaw, Andrea Sherriff

**Affiliations:** ^1^ School of Medicine, Dentistry, and Nursing, College of Medical, Veterinary and Life Sciences University of Glasgow Glasgow UK; ^2^ Scottish Centre for Social Research Edinburgh UK

**Keywords:** child health, dental caries, epidemiology, longitudinal, medical record linkage, socioeconomic factors

## Abstract

**Objective:**

This study aimed to explore the longitudinal impact of changes in household income poverty and area‐based socioeconomic deprivation on dental caries prevalence in early childhood.

**Methods:**

Data from the Growing Up in Scotland (GUS) longitudinal study (2005/6‐2009/10) were linked to dental caries experience data at age 5 from Scotland's National Dental Inspection Programme. Latent Class Analysis identified trajectories of household poverty (income below 60% of the national median) and area‐based deprivation across multiple time points between birth and age 5. Cumulative exposure scores were also calculated, and modified Poisson regression assessed associations between socioeconomic pathways and caries experience in 2893 children.

**Results:**

Children living in persistent household poverty or in socioeconomically deprived areas had the highest caries experience risk compared to children never in poverty or deprivation. Elevated caries risk was also observed in children falling into poverty (aRR = 1.4; 95% CI = [1.1–1.8]) and escaping poverty (aRR = 1.6; 95% CI = [1.3–2.1]). Children moving into more deprived areas had higher caries risk (aRR = 1.6; 95% CI = [1.2–2.2]), while moving out of deprived areas did not increase risk (aRR = 1.1; 95% CI = [0.8–1.7]). Caries risk increased with years spent in household poverty and in deprived areas.

**Conclusions:**

Unstable poverty and downward socioeconomic deprivation mobility were associated with greater caries risk in early childhood, underscoring the importance of considering the duration and persistence of socioeconomic disadvantage in relation to oral health outcomes and should inform early‐years focused policies to address these. Longitudinal data linkage combining representative surveys and routine data is a powerful way to uncover these issues.

## 1 Introduction

The detrimental impact of negative socioeconomic circumstances (SEC) on children's oral health outcomes is clear [1]. Understanding their complex role is crucial for developing and evaluating efficient and effective interventions, with early‐year interventions such as Scotland's Childsmile Programme highlighted as critical for addressing health inequalities [2, 3, 4]. These points are particularly relevant to child dental caries, an early physical marker of general health [5] and the most prevalent non‐communicable disease in the world [6], with stark socioeconomic inequalities existing both between [7] and within countries [8, 9].

Defining an individual's socioeconomic circumstances can be complex, as the relevance of each possible metric varies depending on the focus of the study [10]. In Scotland, monitoring health inequalities has primarily relied on multifaceted area‐based socioeconomic deprivation measure, the Scottish Index of Multiple Deprivation (SIMD) [11]. This assesses levels of deprivation based on multiple indicators across 7 domains [12]. Area‐based measures of deprivation are easier to obtain than individual measures, relying mostly on readily available routine data and home address. These measures are cost‐effective, maintain privacy and are useful for identifying geographic concentrations of need [13]. However, they have limitations, such as overlooking individual socioeconomic experiences. SIMD, for example, has been shown to exhibit low sensitivity in identifying individuals suffering from income poverty [13, 14]. This is vital, as household income poverty has been identified as a recurring key factor in previous studies investigating childhood caries [1, 15]. As of 2022/23, 26% of the children in Scotland were living in relative poverty [16], where individuals lack the minimum income or resources to maintain the average standard in their country of residence, defined by a household receiving less than 60% of the relevant country's median household income.

In addition to the use of individual‐level versus area‐based measures, it is crucial to consider different data collection methods and a variety of analysis techniques when considering socio‐economic circumstances (SEC). For example, cross‐sectional studies versus longitudinal studies and social mobility trajectories versus cumulative effects each present their own strengths and limitations [17, 18], For research on oral health inequalities to shift away from documenting to explaining, and association to causation, the use of longitudinal data are necessary [19]. They allow the exploration of prolonged exposure and social mobility [17], improving understanding of complex mechanisms producing inequalities and helping refine effective interventions. Longitudinal data also help address temporality concerns and avoid the bias common during the interpretation of cross‐sectional analyses of associations [20].

Social mobility has been shown to have a substantial effect on health outcomes. Upward social mobility potentially mitigates the negative health effects associated with low SEC [21], while downward socioeconomic mobility has been shown to lead to worse health outcomes [22, 23, 24]. These findings emphasise the importance of understanding the dynamics over time and lay the foundations for using trajectory analyses in SEC studies [18, 25].

To effectively address dental caries, understanding the role of SEC in shaping health disparities is critical to optimising the impact of public health interventions [26], such as the Childsmile Programme in Scotland [4]. Analysing these disparities demands a temporal approach that integrates multiple measures and metrics. Therefore, the aim of this study is to investigate the impact of early life longitudinal and cumulative exposures to income poverty and area‐based deprivation on caries experience at 5 years.

## 2 Methods

### 2.1 Data

This study utilised a data linkage, longitudinal design, linking data from the Growing Up in Scotland (GUS: 2005/06‐2009/10) study with caries experience data at age 5 from the National Dental Inspection Programme (NDIP) in Scotland. GUS is a longitudinal birth cohort study with a nationally representative sample of 5217 children born in 2004/05 in Scotland. This analysis covers data from sweep 1 (2005/06, age 10 months), sweep 2 (2006/07, age 2), sweep 3 (2007/08, age 3), sweep 4 (2008/09, age 4) and sweep 5 (2009/10, age 5) [27]. The data were collected from a main carer/parent at each sweep, with face‐to‐face surveys carried out in participants' homes by trained social survey interviewers using laptop computers.

The National Dental Inspection Programme (NDIP) database [9] includes an annual survey of oral health outcomes for all Primary 1 school‐year (approximately 5 years old) children attending local authority schools and some public schools. The inspection involves a simple standardised assessment of the mouth and teeth undertaken in the school by trained and standardised community dental teams.

Inclusion was based on consistent participation across survey sweeps, agreeing to data linkage and having a valid NDIP exam from school years 2009/10 and 2010/11 (*n* = 2893). Numbers will still vary for each socio‐economic (SE) variable due to varying item missingness. Complete data for each SE variable and caries experience outcome needed to be included in analyses.

Data linkage was performed by the Electronic Data Research and Innovation Service (eDRIS) at Public Health Scotland (PHS) with strict disclosure protocols. Individual‐level data were linked via Scotland's Community Health Index (CHI) numbers through probabilistic matching based on date of birth, sex and postcode. The linkage agent pseudonymised study‐specific identifiers, allowing for the integration of datasets stored within the National Safe Haven (NSH). Both the NDIP and GUS datasets underwent rigorous data cleaning and quality assurance following standard operating procedures.

### 2.2 Key Variables

Household income poverty status at age 2–5 years old: Measured as income poverty, defined by the respondent's annual income after tax but before housing costs being less than 60% of median equivalised income in Scotland, using the modified Organisation for Economic Co‐operation and Development equivalence scale. This scale assigns weights of 0.67 for the first adult, 0.33 for additional adults aged 15 or over, and 0.2 for children aged 0–14 years, providing a comparable poverty threshold across time and populations [28].

Area‐based deprivation was measured using a mix of the Scottish Index of Multiple Deprivation (SIMD) 2004 (Sweep 1), 2006 (Sweeps 2–4), and 2009 (Sweep 5) at ages 10 months to 5 years [29, 30, 31]. It is based on 31/37/38 indicators in six/seven individual domains of current income, employment, housing, health, education, skills and training, geographic access to services, with crime added as a domain in 2006. SIMD is calculated at data zone level, enabling small pockets of deprivation to be identified. The data zones are ranked from most deprived (1) to least deprived (6505) on the overall SIMD index. The data are then recorded in fifths (SIMD1‐SIMD5), with SIMD1 being the most 20% deprived areas in the population.

The multi‐category variable area‐based deprivation was dichotomised with children living in the 20% most deprived areas versus children living in the 80% least deprived areas to simplify the modelling process by reducing the complexity of the grouping output. This facilitated identifying and interpreting distinct patterns or trajectories.

All calculations were conducted at four or five distinct time points between the ages of 10 months/2 years and 5 years. At each time point, a binary coding system was applied: a value of 1 indicated that the individual was in a low SEC, while a value of 0 indicated otherwise. The exposure variable was quantified as both the trajectory over time (detailed in section 2.3) and the cumulative number of years spent in poverty or deprivation, represented as a factor ranging from 0 to 4 or 5, depending on the total number of time points assessed.

Caries experience at age 5 (yes/no) (Outcome): Defined as the presence of obvious decay which was determined clinically by the presence of decay (caries into dentine), missing (extracted due to decay) or filled deciduous teeth following recognised criteria. This variable was obtained from the National Dental Inspection Programme database [9].

### 2.3 Statistical Analysis

Latent Class Growth Analysis (LCGA) is a statistical tool for analysing trajectory patterns in data over time. LCGA identifies distinct subgroups within a population that share similar developmental trajectories, revealing patterns that might be missed in cross‐sectional studies [32].

In this study LCGA was used to identify longitudinal trajectories of household income poverty from age 2 to age 5 years (4 discrete time points) and area‐based deprivation from 10 months to age 5 (five discrete time points). This discrepancy was caused due to not having access to income data at 10 months of age. LCGA classifies individuals into mutually exclusive latent classes based on patterns in observed categorical variables [33]. Models' parameters were estimated using maximum likelihood with robust standard errors. The best‐fitting model was selected based on the lowest Bayesian Information Criterion (BIC) value, interpretability, and theoretical relevance. Models with 3–5 classes were estimated using the R package ‘poLCA’ [34]. Model adequacy was assessed through posterior probabilities, with cumulative exposure scores calculated [33].

Due to the binary nature of caries experience, a modified Poisson regression model with robust error variances was used to estimate risk ratios and 95% confidence intervals (CI) for caries experience, with those consistently never in poverty or deprivation as the reference categories. The regression models were adjusted for the child's sex and age at the time of the NDIP exam.

Longitudinal Survey weights are used throughout this study. The weights are developed using a model‐based weighting technique where response behaviour is modelled using data from previous sweeps detailed further in GUS Sweep 5 User Guide [27].

The study received ethical approval (College of MVLS, University of Glasgow) and approval from the Public Benefit and Privacy Panel—Health and Social Care (PBPP‐ Public Health Scotland) and the Statistics‐ PBPP (Scottish Government) (reference number: 200170146).

## 3 Results

There were 3621 children in the GUS Birth Cohort 1 present in all sweeps, of which 3275 (90.4%) provided consent for their data to be linked and analysed in the NSH. Those missing (*n* = 346) did not give consent for their survey data to be linked to routine administrative data or were not successfully linked.

There were 51 074 Primary 1 children who had a NDIP record in the same timeframe; of the 3275 children in GUS, 2893 (88.3%) had a valid NDIP exam. This becomes 2875 children after survey weights are applied. For these children, the item response rates for each socioeconomic variable differ. Income Poverty has *n* = 2516 (87.0%) responses, or *n* = 2484 (85.9%) after GUS survey weights are applied. The missing data for Income Poverty did not appear to be socially patterned according to SIMD. The sample characteristics along with the number of children in poverty/deprivation at each age are displayed in Table 1.

**TABLE 1:** Characteristics of sample.

Variable	*n* (%)				
Age (years) at NDIP					
Min	4.110				
Q1	5.311				
Median	5.541				
Q3	5.802				
Max	6.795				
Sex					
Male	1462 (50.9)				
Female	1413 (49.1)				
Caries experience at age 5 based on basic NDIP examination					
Yes	916 (31.8)				
No	1960 (68.2)				
Sweep age	10 months	2 years	3 years	4 years	5 years
SIMD fifths					
1	697 (24.5)	703 (24.7)	697 (24.4)	687 (24.1)	660 (23.1)
2	528 (18.5)	520 (18.2)	505 (17.7)	513 (18.0)	551 (19.3)
3	556 (19.5)	535 (18.8)	542 (19.0)	539 (18.9)	517 (18.1)
4	553 (19.4)	568 (19.9)	558 (19.6)	562 (19.7)	577 (20.2)
5	516 (18.1)	525 (18.4)	549 (19.3)	551 (19.3)	546 (19.1)
Income Poverty [4]					
Yes	N/A	709 (28.6)	667 (26.9)	640 (25.8)	633 (25.5)
No	N/A	1772 (71.4)	1814 (73.1)	1842 (74.2)	1849 (74.5)
Area‐based deprivation: in lowest SIMD 20%					
Yes	697 (24.5)	703 (24.7)	697 (24.4)	687 (24.1)	660 (23.1)
No	2152 (75.5)	2146 (75.3)	2153 (75.6)	2163 (75.9)	2190 (76.9)

*Note:* Small discrepancy in totals due to rounding of survey weight counts. Defined by the respondent's annual income after tax but before housing costs being < 60% of median equivalised income in Scotland, using the modified Organisation for Economic Co‐operation and Development equivalence scale.

Abbreviations: NDIP, National Dental Inspection Programme; SIMD, Scottish Index of Multiple Deprivation.

**FIGURE 1:**
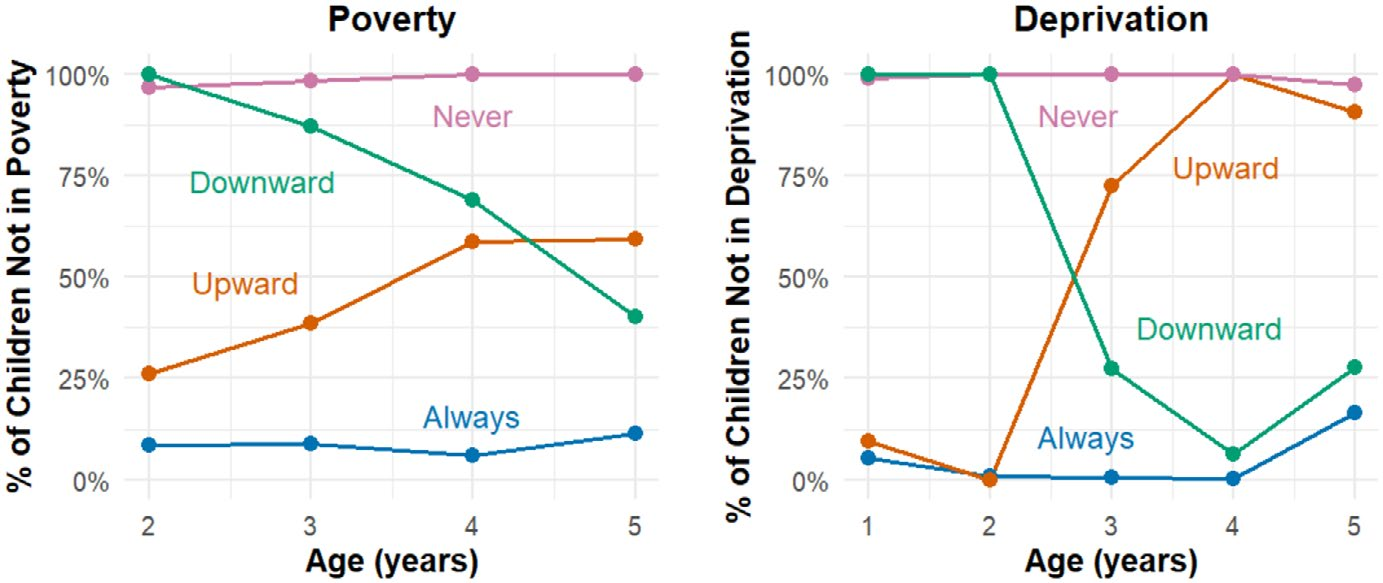
Latent class analysis trajectories of socioeconomic circumstances.

The trajectory groups for both variables are shown below in Figure 1. It can be seen from the findings that some groups are regularly not 100% assigned to either of the binary results for that variable; however, clear patterns still emerge. The SEC levels are defined as ‘Always’ in low SES, ‘Never’ in the low SEC, ‘Upward’ mobility from the low SEC or ‘Downward’ mobility into the low SEC.

Income Poverty has four trajectories: never in income poverty, upward social mobility (escaping Income poverty), downward social mobility (falling into income poverty) and always in income poverty. There are four trajectories present for area‐based deprivation, which can be similarly labelled. Table 2 displays the membership within each trajectory group for each of the variables.

**TABLE 2:** Numbers of children in each poverty/deprivation trajectory.

Outcomes	Poverty (%)	Deprivation (%)
Never	1629 (65.6)	2069 (72.5)
Upward social mobility	199 (8.0)	74 (2.6)
Downward social mobility	145 (5.8)	95 (3.3)
Always	511 (20.6)	615 (21.5)
Total	2484	2853

**FIGURE 2:**
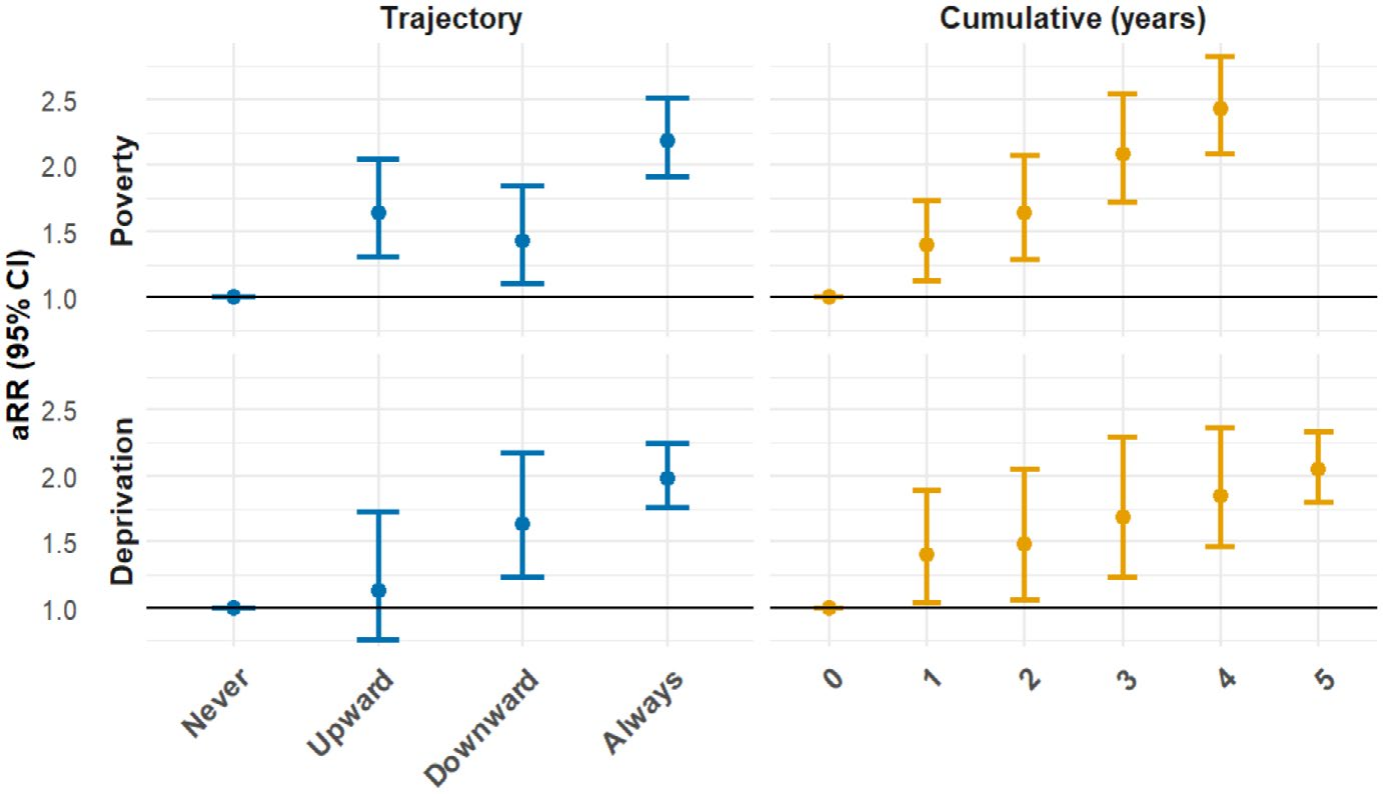
Adjusted risk ratios for caries experience in primary 1 according to of poverty/deprivation trajectories and cumulative affects against caries experience at 5.

Table 2 illustrates that the transitional groups only make up a small proportion of the population; poverty 13.8% (*n* = 344) and deprivation 5.9% (*n* = 169). The proportion of households in poverty and deprivation in every trajectory group also differs at each age (Table S1—Appendix). The associations of these groups with caries experience compared to the risk in the ‘Never’ group for each measure adjusted for sex and age are shown in Figure 2, the values and predictive value of each model as well as the crosstabulations and risk ratios are given in the appendix (adjusted for age at exam and sex) (Table S2—Appendix).

The findings highlight the important impact of poverty trajectories on caries experience risk. Individuals who fall into poverty in the first 5 years have an increased risk (1.43 [1.11–1.85]), while those consistently in poverty face the highest risk (2.19 [1.84–2.60]) compared to those who have never been in poverty. Even after escaping poverty, the risk remains elevated (1.64 [1.33–2.03]). The cumulative effect is also evident, with risk ratios increasing from 1.40 [1.12–1.75] after 1 year of poverty to 2.43 [1.98–2.99] for 4 years, showing the worsening impact of prolonged poverty exposure.

Those who move into the 20% most deprived areas have an increased risk of caries experience (1.63 [1.28–2.07]), and those who always live in the 20% most deprived areas are at highest risk (1.98 [1.64–2.39]). Conversely, those who achieve upward social mobility face a notably lower risk of caries than other deprivation groups (1.13 [0.75–1.72]). The cumulative effect is again clear, with risk ratios increasing from 1.40 [1.04–1.89] after 1 year to 2.05 [1.80–2.33] after 5 years living in the 20% most deprived areas.

As an additional check in the study, the Area‐based Deprivation trajectory model was adjusted for Income Poverty trajectories and vice versa. This was to ensure both socioeconomic variables weren't measuring the same constructs. The key findings were upheld, with income poverty effect once again proving particularly strong. These findings are detailed in the appendix (Table S3).

## 4 Discussion

This study highlights the profound impact of sustained socioeconomic disadvantage, particularly income poverty and area‐based deprivation, on children's dental health. By examining longitudinal patterns of deprivation, this study provides a more comprehensive understanding of how early‐life socioeconomic conditions shape health outcomes, offering insights beyond those of cross‐sectional studies.

There is a well‐established link between early‐life income poverty and poor health outcomes [28, 35, 36], with effects manifesting regardless of other socioeconomic factors [37]. Poverty is believed to be a key factor perpetuating the clear inequalities still present in child health outcomes [36]. These findings are consolidated by this study; with numerous studies already linking income poverty specifically to increased risk of caries experience [38, 39, 40, 41]. This study found that any exposure to poverty—regardless of how many years—elevates caries risk. These findings echo those of Peres, Peres, De Barros and Victora [42] who reported that even brief experiences of poverty in early life are associated with poorer dental health.

The risk of caries increased with each additional year of poverty, clearly demonstrating a cumulative effect. This cumulative effect of poverty was also observed in a previous child caries study [43] and is consistent across other health studies, such as stress responses [44] and child development [45]. The unequivocal and cumulative effect of poverty was also demonstrated by the same GUS cohort when analysing emotional and conduct problems and other health outcomes [28, 46]. These findings align with research showing that cumulative exposure to poverty in childhood can have lasting negative impacts on health, even if socioeconomic circumstances improve later [47, 44].

In addition to income poverty, this study found a strong link between area‐based deprivation and caries experience risk, corroborating previous research [9, 48]. Those living in the 20% most deprived areas consistently displayed higher caries experience risk, with each additional year spent in deprivation further increasing the risk. This supports earlier findings that area‐based deprivation, as a proxy for community‐level inequalities, is a powerful predictor of health disparities [49]. These findings exemplify that prolonged socioeconomic disadvantage, as measured by both income poverty and area‐based deprivation, creates a cumulative risks that exacerbate health inequalities over time, a phenomenon well‐documented in previous health research [50].

The trajectory analysis revealed a novel aspect; children who moved out of the most deprived areas during the study did not experience a notable increase in caries experience risk. This supports the idea that improving socioeconomic conditions can mitigate some negative impacts [21]. In contrast, other studies suggest that early socioeconomic disadvantage has lasting impacts on oral health [51, 52], with early life being a critical period for disease development [28, 42]. This mitigating effect also diverged from the findings on poverty, which may be explained by the differing thresholds required to move out of each circumstance. Escaping poverty could require a minimal financial increase to cross the poverty threshold, whereas reducing area‐based deprivation necessitates substantial changes, such as relocating to a less deprived area or significant improvements in local socioeconomic conditions—changes that are far less common. All this suggests that long‐term exposure to deprivation and social mobility, rather than just a snapshot of current area‐based deprivation status or deprivation at birth, plays a critical role in determining health outcomes. This adds to previous findings drawn from cross‐sectional studies, such as NDIP [9], and provides new insights into the long‐lasting effects of early deprivation and suggests negative effects can be reversed by consequential later improvements measured by a multi‐domain area‐based deprivation. This demonstrates the value of having access to longitudinal data to provide a greater understanding in this area of study.

This study illustrates the importance of understanding the multifaceted effects of living in a less deprived neighbourhood, to evaluate and optimise interventions such as Scotland's Childsmile programme which may target children more appropriately for additional support [4]. However, it also highlights the powerful effect of income poverty alone, suggesting it must be targeted to reduce inequalities in child caries experience; evidence in favour of Scotland's introduction of the child poverty payment in 2022 [53]. The study's longitudinal design enriches our understanding of how both individual and area‐based socioeconomic conditions impact caries experience risk. It highlights the compounding effects of multiple years spent in poverty or deprivation, while also demonstrating that the increased caries risk associated with area‐based deprivation may not persist if children move to less deprived areas. These findings underscore the importance of addressing both the duration and context of socioeconomic disadvantage to effectively reduce health inequalities in children.

### 4.1 Strengths and Limitations

One of the key strengths of this study was the emphasis on capturing the early years of life, recognising that these formative years are crucial in shaping long‐term health outcomes. This study offers long‐term follow‐up, sufficiently large representative sample sizes, and rich baseline covariate data, which collectively help minimise bias and provide a comprehensive view of how early life experiences influence health over time [54].

Another key strength of this study was that the linkage between datasets was robust, with a high linkage rate that did not exclude many records. This, along with the use of high‐quality routine data for the study's outcome and high item response rates for other variables, was an efficient use of time and resources, without any compromise on analysis.

Despite these strengths, the study does have a few limitations. Firstly, this study faced constraints due to a reduced number of time points for poverty status, with area‐based illustrating the clearer trajectories when including a 5th time point at 10 months. This was due to the lack of availability of the data in the National Safe Haven at the time of this study.

Another limitation to consider is that the findings of this study may not extrapolate to other countries and age groups given the different social contexts and healthcare systems.

Furthermore, LCGA presents methodological challenges, including determining the number of latent trajectory classes, selecting appropriate model fit indices, and overcoming convergence issues [33]. Identifying the number of components in a growth mixture model often involves selecting the model with the smallest Bayesian Information Criterion (BIC) value, but convergence problems may arise due to the mathematical complexity of modelling a sample distribution that comprises multiple sub‐distributions [55]. LCGA is susceptible to local solutions, necessitating careful methodological considerations.

A final limitation of this study is the small number of children within the transitional deprivation groups. This is due to few children in the study cohort experiencing a change in SIMD level within the first 5 years of life. This led to wider confidence intervals in the models comparing caries experience across deprivation trajectories, which has reduced the precision of the results.

## 5 Conclusion

In the population studied, unstable income poverty trajectories and area‐based downward socioeconomic mobility were associated with a higher risk of caries experience. These findings underscore the importance of considering the duration and persistence of socioeconomic disadvantage in relation to oral health outcomes. They also emphasise the importance of preventing the first instance of poverty experience to reduce risk. All these findings should inform policies and interventions to address these issues, particularly in the early years. Using longitudinal data linkage to combine representative surveys and routine data is a powerful way to uncover these issues.

## Conflicts of Interest

The authors declare no conflicts of interest.

## Supporting information


Appendix S1.

## Data Availability

Data for this study were obtained from a third party and are not publicly available. We sought approval to access, link, and analyse the study data within the NHS National Safe Haven environment, completing the necessary information governance training. The data generated, linked, and analysed during the study are not publicly available. Public Health Scotland serves as the data controller the dental health data, and researchers may apply for access by contacting phs.edris@phs.scot. The Growing Up in Scotland study was carried out by the Scottish Centre for Social Research and researchers may apply for access through the UK data service.
