# Supplementary material for: Early Life Trajectories of Household Poverty and Area‐Level Deprivation and Childhood Dental Caries: A Longitudinal Data Linkage Cohort Study
Source: Community Dent Oral Epidemiol. 2025 Jun 10;53(5):556–63. doi: 10.1111/cdoe.13051 (PMC12423359; doi:10.1111/cdoe.13051)
Supplement: Supplementary file 1 — Appendix S1. [file CDOE-53-556-s001.docx]

Table S1: Percentage of people in each group in Poverty/Deprivation at each age

|  |  | % Of people in each group in poverty/deprivation | | | | |
| --- | --- | --- | --- | --- | --- | --- |
| Variables | Outcomes | Age 10m | Age 2 | Age 3 | Age 4 | Age 5 |
| Income Poverty | Never In Poverty | N/A | 3.2 | 1.5 | ~0 | 0 |
|  | Upward Social Mobility | N/A | 73.9 | 61.6 | 41.3 | 40.6 |
|  | Downward Social Mobility | N/A | 0 | 12.9 | 31.2 | 59.8 |
|  | Always in Poverty | N/A | 91.4 | 91.1 | 94.2 | 88.5 |
|  |  |  |  |  |  |  |
|  |  |  |  |  |  |  |
| Area-based Deprivation: In Lowest SIMD 20% | Never in 20% | 1.1 | 0 | 0 | 0 | 2.5 |
|  | Upward Social Mobility | 90.6 | 100 | 27.6 | 0 | 9.2 |
|  | Downward Social Mobility | 0 | 0 | 72.5 | 93.7 | 72.3 |
|  | Always in 20% | 94.7 | 99.3 | 99.6 | 99.7 | 83.4 |
|  |  |  |  |  |  |  |

This table illustrates the percentage of each group in poverty/deprivation of each group at each age. E.g 3.2% of all individuals in the ‘Never in Poverty’ Group are in income poverty at Age 2, this decreases to 1.5% by age 3.

Table S2: Risk Ratios for Caries Experience in Primary 1 by Poverty/Deprivation Trajectories and Cumulative Effects at Age 5 year

| Variables | Outcomes | No caries Experience n (%) | Caries Experience n (%) | RR (95% CI) | P-value | AUC [95% CI] |
| --- | --- | --- | --- | --- | --- | --- |
| Income Poverty | Never In Poverty | 1246 (76.5) | 383 (23.5) | 1 (ref) |  | 0.611 [0.585, 0.637] |
|  | Upward Social Mobility | 123 (61.8) | 76 (38.2) | 1.64 (1.31, 2.05) | <0.001 |  |
|  | Downward Social Mobility | 96 (66.2) | 49 (33.8) | 1.43 (1.11, 1.84) | 0.005 |  |
|  | Always in Poverty | 247 (48.3) | 264 (51.7) | 2.19 (1.91, 2.51) | <0.001 |  |
|  |  |  |  |  |  |  |
| Income Poverty: Years in Poverty | 0 | 1156 (77.5) | 336 (22.5) | 1 (ref) |  | 0.622 [0.596, 0.647] |
|  | 1 | 169 (68.7) | 77 (31.3) | 1.40 (1.13, 1.73) | 0.002 |  |
|  | 2 | 114 (63) | 67 (37) | 1.64 (1.29, 2.08) | <0.001 |  |
|  | 3 | 116 (52.7) | 104 (47.3) | 2.09 (1.72, 2.54) | <0.001 |  |
|  | 4 | 157 (45.1) | 191 (54.9) | 2.43 (2.09, 2.82) | <0.001 |  |
|  |  |  |  |  |  |  |
| Area-based Deprivation: In Lowest SIMD 20% | Never in Deprivation | 1533 (74.1) | 536 (25.9) | 1 (ref) |  | 0.603 [0.579, 0.627] |
|  | Upward Social Mobility | 52 (70.3) | 22 (29.7) | 1.13 (0.75, 1.72) | 0.6 |  |
|  | Downward Social Mobility | 55 (57.9) | 40 (42.1) | 1.63 (1.23, 2.17) | <0.001 |  |
|  | Always in Deprivation | 300 (48.8) | 315 (51.2) | 1.98 (1.76, 2.24) | <0.001 |  |
|  |  |  |  |  |  |  |
| Area based Deprivation: Years in Lowest SIMD 20% | 0 | 1466 (74.6) | 498 (25.4) | 1 (ref) |  | 0.611 [0.588, 0.635] |
|  | 1 | 75 (64.7) | 41 (35.3) | 1.40 (1.04, 1.89) | 0.027 |  |
|  | 2 | 52 (62.7) | 31 (37.3) | 1.48 (1.06, 2.05) | 0.020 |  |
|  | 3 | 44 (57.1) | 33 (42.9) | 1.68 (1.23, 2.29) | 0.001 |  |
|  | 4 | 65 (52.4) | 59 (47.6) | 1.85 (1.46, 2.36) | <0.001 |  |
|  | 5 | 237 (48.5) | 252 (51.5) | 2.05 (1.80, 2.33) | <0.001 |  |

1. RR – Risk Ratio (Adjusted for Age at NDIP exam and sex)
2. AUC – Area-under the curve
3. CI – Confidence Interval

Table S3: Adjusted Risk Ratios for Caries Experience in Primary 1 by Poverty/Deprivation Trajectories and Cumulative Effects at Age 5 years

| Variables | Outcomes | RR (95% CI) | P-value | AUC [95% CI] |
| --- | --- | --- | --- | --- |
| Income Poverty | Never In Poverty | 1 (ref) |  | 0.637 [0.612, 0.662] |
|  | Upward Social Mobility | 1.54 (1.24, 1.91) | <0.001 |  |
|  | Downward Social Mobility | 1.40 (1.13, 1.74) | 0.002 |  |
|  | Always in Poverty | 1.88 (1.61, 2.20) | <0.001 |  |
|  |  |  |  |  |
|  |  |  |  |  |
| Area-based Deprivation: In Lowest SIMD 20% | Never in Deprivation | 1 (ref) |  | 0.637 [0.612, 0.662] |
|  | Upward Social Mobility | 0.92 (0.58, 1.45) | 0.7 |  |
|  | Downward Social Mobility | 1.45 (1.04, 2.03) | 0.029 |  |
|  | Always in Deprivation | 1.57 (1.36, 1.81) | <0.001 |  |

1. RR – Risk Ratio (Adjusted for Age at NDIP exam and sex)
2. AUC – Area-under the curve
3. CI – Confidence Interval
